# A review of synthetic cathinones emerging in recent years (2019–2022)

**DOI:** 10.1007/s11419-022-00639-5

**Published:** 2022-09-15

**Authors:** Patryk Kuropka, Marcin Zawadzki, Paweł Szpot

**Affiliations:** 1 Department of Forensic Medicine, Wroclaw Medical University, 4 J. Mikulicza‑Radeckiego Street, 50345 Wroclaw, Poland; 2 Institute of Toxicology Research, 45 Kasztanowa Street, Lower Silesia Province, 55093 Borowa, Poland

**Keywords:** Synthetic cathinones, Novel psychoactive substances, *N*-Ethylhexedrone analogs, Early warning systems, Drug user forums

## Abstract

**Purpose:**

The emergence of novel psychoactive substances (NPS) has been being a continuous and evolving problem for more than a decade. Every year, dozens of new, previously unknown drugs appear on the illegal market, posing a significant threat to the health and lives of their users. Synthetic cathinones are one of the most numerous and widespread groups among NPS. The purpose of this work was to identify and summarize available data on newly emerging cathinones in very recent years.

**Methods:**

Various online databases such as PubMed, Google Scholar, but also databases of government agencies including those involved in early warning systems, were used in search of reports on the identification of newly emerging synthetic cathinones. In addition, threads on various forums created by users of these drugs were searched for reports on the effects of these new substances.

**Results:**

We have identified 29 synthetic cathinones that have been detected for the first time from early 2019 to mid-2022. We described their structures, known intoxication symptoms, detected concentrations in biological material in poisoning cases, as well as the countries and dates of their first appearance. Due to the lack of studies on the properties of the novel compounds, we compared data on the pharmacological profiles of the better-known synthetic cathinones with available information on the newly emerged ones. Some of these new agents already posed a threat, as the first cases of poisonings, including fatal ones, have been reported.

**Conclusions:**

Most of the newly developed synthetic cathinones can be seen as analogs and replacements for once-popular compounds that have been declining in popularity as a result of legislative efforts. Although it appears that some of the newly emerging cathinones are not widely used, they may become more popular in the future and could become a significant threat to health and life. Therefore, it is important to continue developing early warning systems and identifying new compounds so that their widespread can be prevented.

## Introduction

The phenomenon of new psychoactive substances (NPS) is an ongoing problem in modern forensic toxicology. One of the most important groups among NPS are synthetic cathinones, which are derivatives of cathinone, a natural alkaloid found in the shrub *Catha edulis*. This substance has been shown to have psychoactive properties, with a widespread centuries-old tradition among the Arabian Peninsula population of chewing the leaves of this plant for recreational purposes [1–3]. The last decade has been characterized by a remarkable dynamism of change in the illicit drug market with the constant introduction of new substances synthesized to circumvent regulation. This phenomenon has also applied to synthetic cathinones [4–6]. Due to the highly variable nature of the NPS phenomenon, the latest emerging derivatives are largely unknown in terms of their pharmacological properties and toxicity; hence, most of the relevant information is obtained from reported cases of both fatal and non-fatal intoxication [7–9].

We aimed to identify the newly emerged synthetic cathinones in 2019–2022 and to describe their chemical structure, pharmacological properties, toxicities, case reports of fatal poisonings and described symptoms of intoxication. We used the following websites of institutions and government agencies involved in early warning systems and the identification of these compounds: the United Nations Office on Drugs and Crime (UNODC); the European Monitoring Centre for Drugs and Drug Addiction (EMCDDA); the World Health Organization (WHO); the National Forensic Laboratory Information System (NFLIS), a program of the Drug Enforcement Administration (DEA); the NPS Discovery database at the Center for Forensic Science Research and Education (CSFRE); the Scientific Working Group for the Analysis of Seized Drug (SWGDRUG) database and the European project RESPONSE database at the Slovenian National Forensic Laboratory (NFL). For some substances, we have included reports from users of these drugs published on drug user forums (mainly Reddit). For some derivatives, especially those that apparently have not been widely used, the available data are often limited mostly to information on the identification of the compound reported to one of the international early warning systems such as the EMCDDA Early Warning System (EWS). In some cases, readily available analytical data are limited to data from manufacturers of reference materials of specific compounds. For some drugs, analytical and epidemiological data are very limited, while for others there are no reliable sources of information, with only threads on various forums created by users of these substances, so-called „psychonauts”. Gray literature websites and drug user forums can be a valuable source of knowledge about the emergence of new compounds on the drug market, often providing a much quicker indication of changes and trends in the appearance of new substances on the market [10–13]. In addition, for the newest compounds, given the absence of any other sources due to the lack of any pharmacological studies, Internet forums may provide the only knowledge of the routes of administration, desired and side effects and doses [10, 14]. Particularly during the COVID-19 pandemic, such forums have become increasingly important for marketing and information about NPS [13]. However, it is worth keeping in mind that despite global access, forums may not reflect the global situation, but rather the local status, as the dominant proportion of users may only come from a specific region of the world [10]. Furthermore, very often NPS users are not aware of what drug they are taking, and the actual subjective symptoms described may relate to drugs other than those indicated by the user or may be deliberately communicated for some reason to be misleading and untrue. We have described a time period that coincides with the COVID-19 pandemic, which has affected the landscape of the NPS market. Both the production and distribution of these drugs have been disrupted, which has partly affected users' access to these drugs. Restrictions on social life and relations and the absence of large-scale events such as music festivals may have reduced or shifted demand for other types of substances [1–4].

## Newly emerging synthetic cathinones in 2019–2022

**Fig. 1:**
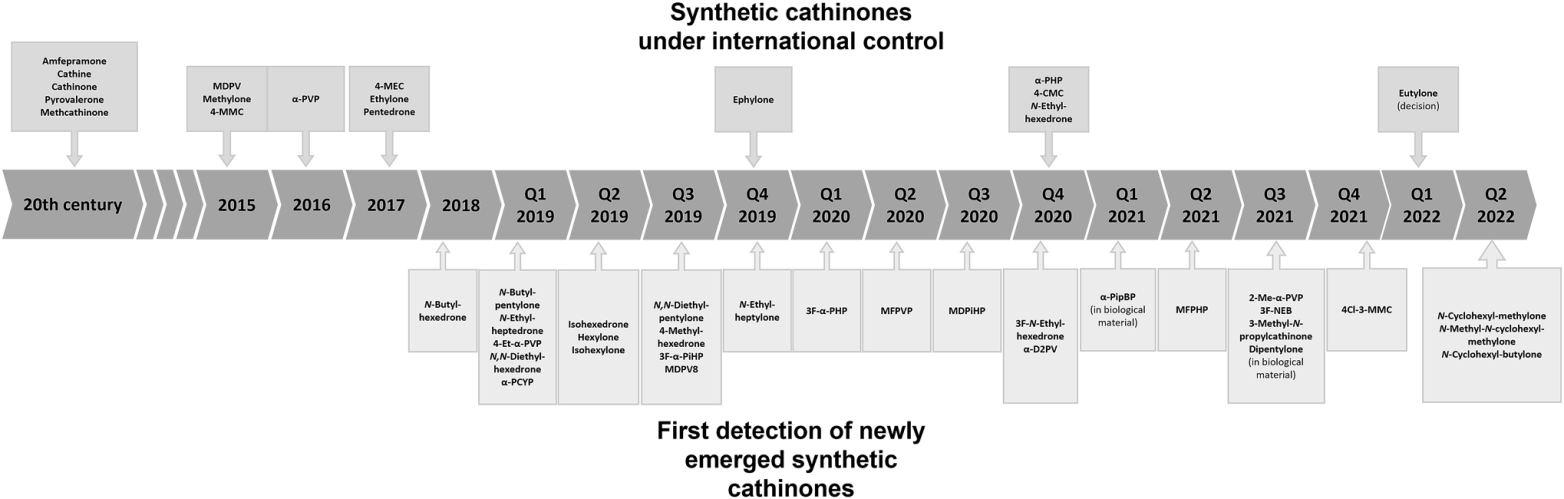
Time line of discoveries of newly emerging synthetic cathinones for the first time. Below the line, the names of 29 newly emerging synthetic cathinones (their chemical structures, see Fig. 7); above the line, international scheduling decisions on the drugs

We have identified a total of 29 synthetic cathinones that were detected for the first time during 2019–2022 (Fig. 1, drug names given just below the time line). In November 2018, *N*-butylhexedrone (2-(butylamino)-1-phenylhexan-1one) was seized for the first time in Russia in a shipment originating from China [15]. However, the first analytical data in the public domain appeared at the beginning of 2019 [16]. At that time, this derivative was also seized for the first time in the European Union (EU), including Poland [17]. In February 2019, *N*-butylpentylone (1-(1,3-benzodioxol-5-yl)-2-(butylamino)pentan-1-one) was seized for the first time in the USA [18]. In that year, it was one of the most reported synthetic cathinones in the USA [19], and it was also identified in Europe, including Hungary [20] and Switzerland [21], as well as in China and Singapore [22]. Similarly, in Hungary *N*-ethylheptedrone (2-(ethylamino)-1-phenylheptan-1-one) was identified for the first time in February 2019 [23]. It gained considerable attention and in that same year detected in 22 biological samples across south-west Hungary [20]. 4-Ethyl-α-PVP (1-(4-ethylphenyl)-2-(pyrrodin-1-yl)pentan-1-one) was detected in Hungary in March 2019 [23], but to the best of our knowledge no data on its prevalence is available and little analytical data exist online for this derivative [24], which may suggest its limited prevalence. The other derivative for which there is little available data is *N,N*-diethylhexedrone (2-(diethylamino)-1-phenylhexan-1-one), which was first detected in the USA, with analytical data published in March 2019 [25]. Another cathinone detected for the first time in the same month in the USA was α-PCYP (2-cyclohexyl-1-phenyl-2-pyrrolidin-1-yl-ethan-1-one) [26]; in Europe, the first report was made in January 2020 in Sweden [23]. Because of its relatively unconventional structure as compared with other synthetic cathinones, this compound has been widely discussed on drug user forums [27]. It is worth noting that α-PCYP had been synthesized in the laboratory in 2015 and tested in systematic structure–activity relationship (SAR) studies as one of the derivatives of pyrrolidine cathinone derivatives [28]. Yet another derivative detected in the first half of 2019 was isohexedrone (4-methyl-2-(methylamino)-1-phenylpentan-1-one) detected in Sweden in April [23]; however, we found almost no information on this drug regarding analytical and epidemiological data or pharmacological properties [29]. In a similar time period, hexylone (1-(1,3-benzodioxol-5-yl)-2-(methylamino)-hexan-1-one) and isohexylone (1-(1,3-benzodioxol-5-yl)-4-methyl-2-(methylamino)pentan-1-one) both structurally similar to isohexedrone were first detected in May in Germany and June in the UK [23]. Hexylone was seized in the USA for the first time in March 2020, and its analytical data have been published [30]. On the other hand, data on isohexylone are highly limited [31]. In August 2019, *N,N*-diethylpentylone (1-(1,3-benzodioxol-5-yl)-2-(diethylamino)pentan-1-one), an *N,N*-diethyl derivative of the then popular ephylone (1-(1,3-benzodioxol-5-yl)-2-(ethylamino)pentan-1-one) was added to the NFLIS-Drug database [32]. *N,N*-Diethylpentylone was reported for the first time in Europe (Spain) over a year later in July 2020 [23]. However, available information on this derivative is very limited [33], and it seemingly has not been commented on in the forums at all, which may suggest that this derivative existed, for example, only as an impurity (adulterant) resulting from the synthesis of another similar compound such as the popular mono-*N*-ethyl derivative. Also in August 2019, new synthetic cathinone 4-methylhexedrone (2-(methylamino)-1-(4-methylphenyl)-1-hexanone) was identified for the first time in the EU [23]. It was discussed, among others, on the Polish drug user forum [34], but apart from reports from users, information about this derivative is limited [35].

In the second half of 2019, three more novel derivatives were detected in Sweden: 3F-α-PiHP (1-(3-fluorophenyl)-4-methyl-2-(pyrrolidin-1-yl)pentan-1-one) in August, MDPEP also known as MDPV8 (1-(1,3-benzodioxol-5-yl)-2-(pyrrolidin-1-yl)-heptan-1-one) in September, and *N*-ethylheptylone (1-(1,3-benzodioxol-5-yl)-2-(ethylamino)heptan-1-one) in October [23]. Analytical and pharmacological data on 3F-α-PiHP were very limited, with some individual threads on the web on psychonauts forums [36]. It seems that *N*-ethylheptylone has not been discussed at all on the forums, and some analytical data have been published by the Hungarian Institute for Forensic Science in the Project RESPONSE database [37]. Far more data are available for MDPV8; it has been discussed quite extensively on psychonaut forums since May 2019 [27]. Between May 2020 and April 2021, it was found at least twice in toxicology cases in postmortem biological samples in the USA [38]. Additionally, according to the NFLIS-drug database, it was one of the most frequently reported synthetic cathinones in the USA in 2020 [39, 40] as well as the first half of 2021 [41]. Another cathinone detected for the first time in Sweden in January 2020 was 3F-α-PHP (1-(3-fluorophenyl)-2-(pyrrolidin-1-yl)-hexan-1-one) [23], but similarly to its isomer 3F-α-PiHP, information on this derivative is very limited [42]. Meanwhile, a few months later, in April 2020, the structural analog of 3F-α-PHP, namely 4F-3-methyl-α-PVP, also known as MFPVP (1-(4-fluoro-3-methylphenyl)-2-(pyrrolidin-1-yl)pentan-1-one), was first detected in Sweden [23], and in August, it was discovered for the first time in the USA [43], where it soon became significantly widespread [44]. There had been extensive discussion of it on psychonaut forums already in May 2020 [27]. According to NFLIS-Drug Public Data, it was detected dozens of times in Florida during 2020 [40], and during the first half of 2021 this number was as high as 80 reports [41]. However, according to the latest DEA Threat Report Mid-Year 2021, out of 105 cathinone identifications, only one case involved MFPVP [45]. In April 2021, a higher homolog of MFPVP—MFPHP also known as 4F-3-methyl-α-PHP (1-(4-fluoro-3-methyl-phenyl)-2-pyrrolidin-1-yl-hexan-1-one)—was detected in Sweden for the first time [46]. However, apart from the information on its detection, there is limited data available on this compound. The last synthetic cathinone we identified that has simultaneously a methyl and halogen substituent on the phenyl ring is 4Cl-3MMC, also known as 4-chloro-3-methylmethcathinone (1-(4-chloro-3-methylphenyl)-2-(methylamino)propan-1-one), but information on this compound is limited only to the report of the first detection in August 2021 in Sweden [46]. Two other derivatives that were detected for the first time in Sweden in August and October 2020 were MDPiHP (1-(1,3-benzodioxol-5-yl)-4-methyl-2-pyrrolidin-1-yl-pentan-1-one) and 3F-*N*-ethylhexedrone (2-(ethylamino)-1-(3- fluorophenyl)hexan-1-one) [23]. Besides information about the detection and data from the manufacturer of the analytical reference standard, there are no further available data [47, 48]. In December 2020, α-D2PV (1,2-diphenyl-2-(pyrrolidin-1-yl)ethan-1-one) [46, 49] was reported for the first time as part of the European Project RESPONSE, and in April 2021 in the USA, the compound was added to the NFLIS-Drug database [32]. This derivative, due to its unique structure—similarly to the case of α-PCYP—has been widely commented on in drug user forums [50].

In early 2021, α-piperidinobutiophenone, also known as α-PipBP (1-phenyl-2-(piperidin-1-yl)butan-1-one), was identified for the first time in biological material in a urine sample by the US DEA in collaboration with the University of California San Francisco Clinical Toxicology and Environmental Biomonitoring (CTEB) Laboratory. However, this compound had already been identified previously in the USA in seized material in August 2017 [51], whereas in Europe it was first detected as early as 2012 in Spain [52]. In the second half of 2021 in Sweden, two more cathinone derivatives were detected for the first time: 2-Me-α-PVP (1-(2-methylphenyl)-2-(pyrrolidin-1-yl)pentan-1-one) in July and 3F-NEB (2-(ethylamino)-1-(3-fluorophenyl)butan-1-one) in August [46]. Both derivatives have been commented on extensively on drug user forums; however, apart from that there is little data available for these derivatives [53, 54]. At a similar time, another synthetic cathinone, 3-methyl-*N*-propylcathinone also known as 3-methyl-α-propylaminopropiophenone (2-(propylamino)-1-(3-methylphenyl)-1-propanone) was detected for the first time. However, the data regarding this derivative is limited only to information about the detection of this compound in August 2021 in Hungary [46].

Furthermore, in late 2021 and early 2022, threads appeared on drug user forums regarding an alleged novel derivative sold online under the street name cyputylone, which is supposed to be the compound *N*-cyclohexylmethylone (1-(benzo-1,3-dioxol-5-yl)-2-(cyclohexylamino)propan-1-one). Moreover, the Society of Forensic Toxicologists (SOFT) NPS Committee in collaboration with the CFSRE included *N*-cyclohexylmethylone together with its *N*-methyl derivative *N*-cyclohexyl-*N*-methylmethylone (1-(benzo-1,3-dioxol-5-yl)-2-(cyclohexyl(methyl)amino)propan-1-one) in their Recommended Scope for NPS Testing in the United States for the fourth quarter (Q4) 2021, providing suggested cutoff concentrations or reporting limits of 1—10 ng/mL for both compounds [55]. Most recently, in May 2022, the CFSRE also provided the first information regarding the detection of *N*-cyclohexylmethylone in drug material by the Indianapolis-Marion County Forensic Services Agency and the Miami Dade Police Department [56], and a month later the first detection of its structural analog, *N*-cyclohexylbutylone, was also reported [57]. Therefore, it is likely that these substances had already appeared on the illicit market and will begin to be identified more frequently.

Despite the emergence of many novel synthetic cathinones during 2019—2021, the most prevalent derivatives in this time period were those that had been known previously. *N*-Ethylpentylone, also known as ephylone (1-(1,3-benzodioxol-5-yl)-2-(ethylamino)pentan-1-one), was first detected in 2014, but it was not until 2016 that it began to be reported more frequently, eventually becoming one of the most prevalent synthetic cathinones in 2019 [58–62]. Following temporary scheduling in 2018 in the USA [63] and international scheduling a year later [64] its prevalence started to decline significantly and it was quickly replaced on the market by *N*-ethylbutylone, also known as eutylone (1-(1,3-benzodioxol-5-yl)-2-(ethylamino)butan-1-one), which shares a similar structure [65–67]. Many years have passed between the first detection of eutylone in Poland in 2014 [68] and when it became one of the most commonly identified NPS in 2019—2021 [39, 41, 67]. Taking this as an example, it is reasonable to assume that some of the derivatives first identified in 2019—2021 may be more widely distributed in the future. Another synthetic cathinone that has been known for a long time, but which has only gained popularity in 2019—2021, is 3,4-methylenedioxy-*N*-benzylcathinone, also known as benzylone (1-(1,3-benzodioxol-5-yl)-2-(benzylamino)propan-1-one). This compound was first reported to the EMCDDA back in 2010 [69], but between 2011 and 2018, there were 33 seizures of this drug. In 2019 alone, this number nearly tripled, with benzylone beginning to be identified in countries where it had not been present in the past [70–72].

Finally, data from the USA show that as a result of international scheduling of eutylone, dipentylone (1-(1,3-benzodioxol-5-yl)-2-(dimethylamino)pentan-1-one) has become increasingly common as a replacement. Although this derivative had been detected already in 2014 in Sweden [68], it did not gain much popularity then, and only started to be detected again between 2019 and 2021 [73–75]. Moreover, between October and December 2021, dipentylone was the second most reported cathinone to NFLIS-Drug [76]. According to the CFSRE data, by March 2022 it had been detected in 32 toxicology cases, including 26 postmortem investigations [77]. One case report of death due to polydrug abuse including dipentylone is also available in the literature [62].

## Legal status and legal approaches

The NPS phenomenon remains a considerable challenge due to its evolving nature. The range of compounds currently on the drug market is constantly changing, with new and previously unknown substances entering the market every year. Despite the implementation of regulatory countermeasures and the development of extensive early warning systems and their adaptation to the current situation, NPS including synthetic cathinones are still available and widespread [78, 79]. One of the potential reasons for the continuous phenomenon of NPS is the lack of international consensus on the legal control of these drugs despite the existence of a common basis for UN member countries, namely the three International Drug Control Conventions: the 1961 United Nations Single Convention on Narcotic Drugs, the 1971 United Nations Convention on Psychotropic Substances, and the 1988 United Nations Convention Against Illicit Traffic in Narcotic Drugs and Psychotropic Substances. Different legislative approaches in each country affect local drug market appearance and control, and may contribute to different dynamics of developing NPS situations in different regions of the world [80, 81]. Some countries base their drug policy on individual listing, some on generic legislation or analog control, and in many countries there is a hybrid system drawing on solutions from different legislative approaches [81–84]. In 2013, the UNODC established an Early Warning Advisory (EWA) as a response at the global level to the increasing prevalence of NPS [85]. Since its launch in 2013 until the beginning of 2022, a total of 136 countries had reported a total of over 1100 individual substances [86]. Furthermore, at the European level, EMCDDA had monitored around 830 NPS at the end of 2020, 46 of which had been detected for the first time in Europe in 2020 [87]. In recent years, the number of substances reported in Europe has remained more or less constant at 400 compounds each year. In 2019, synthetic cathinones (156 compounds) were the second most numerous group after synthetic cannabinoids (209 compounds); these categories accounted for almost 60% of the number of seizures in EU Member States [87].

Globally, the Commission on Narcotic Drugs (CND) under the three International Drug Control Conventions is empowered to decide on the scope of control substances and their scheduling (Fig. 1, drugs given just above the time line). The first of the synthetic cathinones included in the original list of the Convention on Psychotropic Substances of 1971 was amfepramone, also known as diethylpropion (2-(diethylamino)-1-phenylpropan-1-one), classified as in Schedule IV. Cathine (2-amino-1-phenylpropan-1-ol) was listed in 1985 (Schedule III), followed a year later by cathinone (2-amino-1-phenylpropan-1-one) (Schedule I), along with pyrovalerone (1-(4-methylphenyl)-2-(1-pyrrolidinyl)pentan-1-one) (Schedule IV). Ten years later, methcathinone (2-(methylamino)-1-phenylpropan-1-one) (Schedule I) was added in 1995. There are currently 17 synthetic cathinones scheduled at the global level; the majority of these were added after 2015 [88]. MDPV (1-(1,3-benzodioxol-5-yl)-2-(pyrrolidin-1-yl)pentan-1-one), methylone (1-(1,3-benzodioxol-5-yl)-2-(methylamino)propan-1-one), and 4-MMC (2-methylamino-1-(4-methylphenyl)propan-1-one) were included in 2015. A year later, α-PVP (1-phenyl-2-(1-pyrrolidinyl)-1-pentanone) was added. In 2017, three more were included: 4-MEC (2-ethylamino-1-(4-methylphenyl)propan-1-one), pentedrone (1-phenyl-2-(methylamino)pentan-1-one) and ethylone (1-(1,3-benzodioxol-5-yl)-2-(ethylamino)propan-1-one) [88]. Ephylone was scheduled in 2019, followed by three more in 2020: α-PHP, 4-CMC (1-(4-chlorophenyl)-2-(methylamino)-1-propanone), and *N*-ethylhexedrone (2-(ethylamino)-1-phenylhexan-1-one). Most recently, eutylone was listed in 2022.

To make the scheduling process more efficient and to adapt it to the regional situation, many countries have created their own schedules. For example, in the USA, there is the Controlled Substances Act (CSA), which contains federal regulations that form the basis for five lists of controlled substances. This classification is based on the addictive potential, harmfulness, and potential therapeutic value of these substances. To make the decision-making process even faster, temporary scheduling within the CSA is also possible. Indeed, in 2021 temporary scheduling was extended by another year for six synthetic cathinones that had been subject to temporary scheduling in 2019 [89], namely *N*-ethylhexedrone, α-PHP, 4-MEAP (2-(ethylamino)-1-(4-methylphenyl)-1-pentanone), MPHP (2-(pyrrolidin-1-yl)-1-(p-tolyl)hexan-1-one), PV8 (1-phenyl-2-pyrrolidin-1-ylheptan-1-one), and 4-Cl-α-PVP (1-(4-chlorophenyl)-2-(1-pyrrolidinyl)-1-pentanone). Permanent scheduling of these compounds, their salts, and their isomers has also been proposed [89]. However, these substances had been known in the USA long before they came under scheduling. According to NFLIS data, the first one was detected in the seized drug evidenced as early as 2012 (MPHP) and the last one in 2016 (*N*-ethylhexedrone) [89]. This demonstrates that in such systems it can take up to several years from the identification of an agent to the introduction of control procedures for the drug [88]. None of the newly emerged synthetic cathinones we identified in 2019–2021 was covered by individual scheduling at the global level, or by the CSA. Structurally, most of the newly emerged cathinones that we identified were analogs of popular compounds that have previously been covered by scheduling, illustrating the link between innovation in the NPS market and proposed legislative solutions, and at the same time making these compounds eligible for scheduling in countries with generic legislation or analog control.

The number of newly emerged NPS on the global market has dropped each year, from 163 in 2013 to 71 in 2019. In developed countries, generic laws and a system based on effective control of analogs have a positive effect, but poorer countries with less effective control systems may be vulnerable to the growth of the NPS problem. However, the production situation developed over the same period in Central and South America from 60 kg secured in 2015 to 320 kg in 2019, and in Africa from less than 1 kg in 2015 to 828 kg in 2019 [90]. Such an increase can only partly be explained by the increase in detection capability of these agents in these areas of the world. Additionally, globally there is great diversity in the scale of the NPS problem. Although globally more than 1 100 individual NPS have been reported to the UNODC EWA system by a total of 136 countries, only 12 of these countries have reported more than 300 compounds to the system (the USA and Sweden have reported the most, with more than 500 compounds), and as many as 90 countries have reported less than 50 individual NPS [86, 91].

## Chemistry

Cathinone, a natural alkaloid, is a β-keto derivative of amphetamine. Together with amphetamines, synthetic cathinones can be classified as phenylethylamines that possess psychostimulant effects, and cathinone derivatives themselves can be described as β-keto phenylethylamines [2, 92]. The basic structure of cathinone consists of a phenyl ring and an attached aminoalkyl chain with a carbonyl group at the beta position [2, 93]. Cathinone derivatives are formed by substitution of the cathinone skeleton at several key positions: at the aromatic ring, the alkyl side chain, and the nitrogen atom in the amino group. Typical substituents are straight and branched alkyl chains as well as halogens. Substitutions can result in a countless number of derivatives, most of which can be assigned to four main structural groups [2, 94]. The first and simplest of them consists of cathinone derivatives substituted with an alkyl group at the nitrogen atom, and the second group, apart from a possible *N*-alkyl substitution, is characterized by substitution of the phenyl ring with a 3,4-methylenedioxy group. The cathinone derivatives in this group structurally and partially pharmacologically resemble 3,4-methylenedioxymethamphetamine (MDMA) [2, 92, 94]. The third group is distinguished by a tertiary amine group in the form of a pyrrolidine ring, while the fourth is a combination of the second and third groups. Derivatives of this group simultaneously have a substituted phenyl ring with a 3,4-methylenedioxy group and an amine in the form of a pyrrolidine ring. In the first and third group, the presence of alkyl or halogen substituents at different positions of the aromatic ring is also possible, and in all groups the alkyl side chain can be characterized by different lengths and branching.

**Fig. 2:**
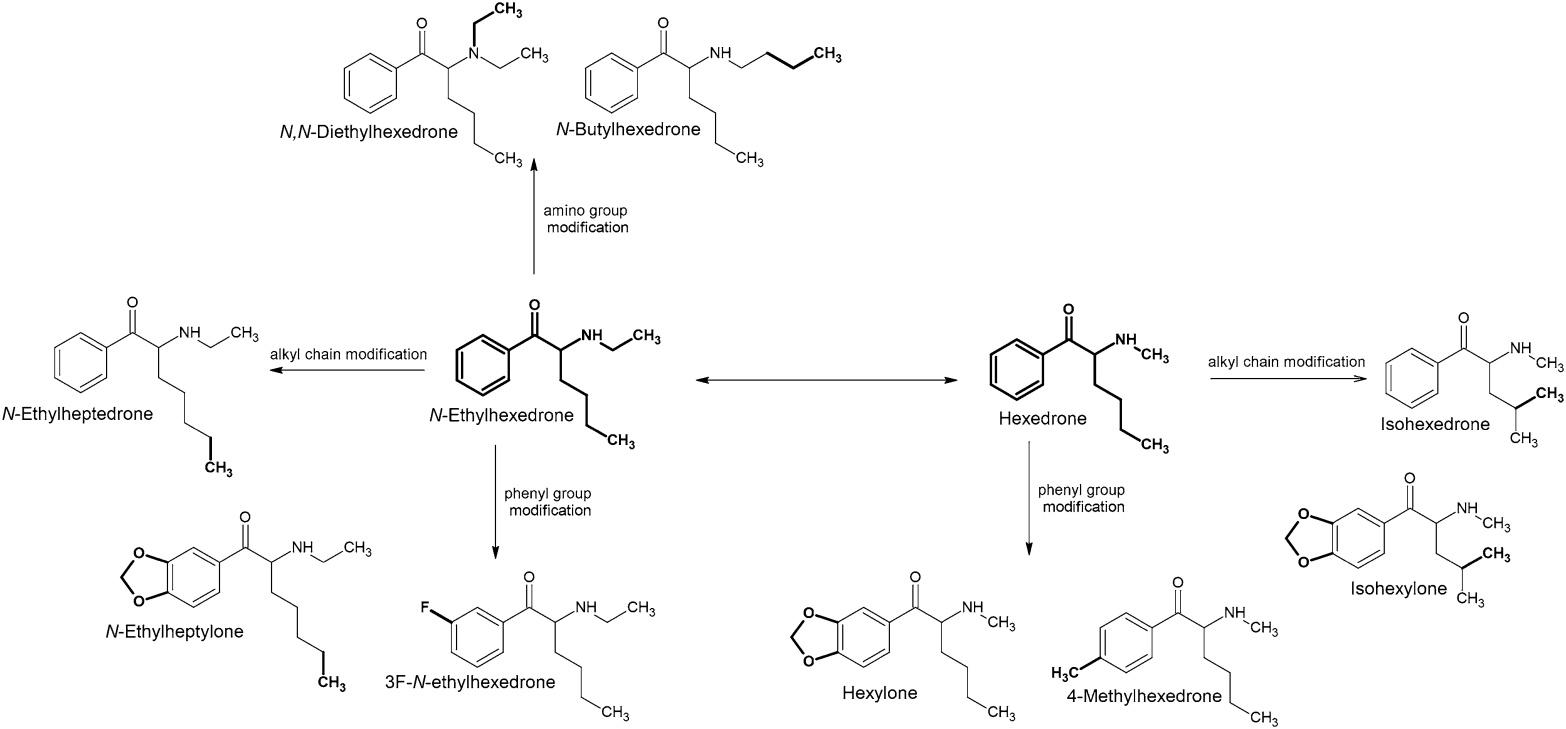
Structures of *N*-ethylhexedrone analogs

The main cause for the emergence of new derivatives and changes in the prevalence of those already present is the introduction of changes in their regulatory status. As a result of scheduling, analogs and isomers of popular compounds that have been banned are rapidly entering the market. *N*-Ethylhexedrone, known since 2016, was quite popular in many countries in 2017 and 2018 [20, 95], and many of the derivatives first identified in 2019–2021 can be considered simple structural alterations of *N*-ethylhexedrone (Fig. 2). Modifications within the amino group have led to the formation of two derivatives. Elongation of the *N*-alkyl chain gave rise to *N*-butylhexedrone, and addition of a second *N*-ethyl chain resulted in the derivative *N,N*-diethylhexedrone. The phenyl ring modification in the form of substitution of a fluorine atom in the *meta* position resulted in the formation of 3F-*N*-ethylhexedrone. On the other hand, elongation of the side alkyl chain of *N*-ethylhexedrone by one carbon atom resulted in formation of *N*-ethylheptedrone, and simultaneous modification of the phenyl ring by the presence of 3,4-methylenedioxy resulted in the formation of *N*-ethylheptylone.

**Fig. 3:**
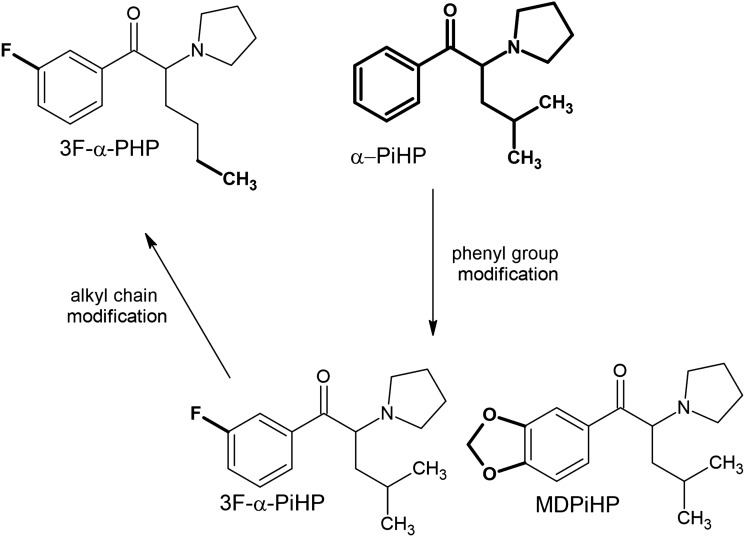
Structures of α-PiHP analogs

The *N*-methyl analog of *N*-ethylhexedrone, namely hexedrone (2-(methylamino)-1-phenylhexan-1-one), has been known since 2014 [96] and represents the starting point for a series of further novel derivatives (Fig. 2). Hexedrone substitutions within the phenyl ring have resulted in the formation of two derivatives: 4-methylhexedrone formed by substitution at the *para* position with a methyl group, while the presence of a 3,4-methylenedioxy group resulted in hexylone and its analog, isohexylone, which is distinguished by a branched alkyl side chain. The same branched alkyl side chain can be found in the hexedrone analog isohexedrone, as well as in α-PiHP (4-methyl-1-phenyl-2-pyrrolidin-1-yl-pentan-1-one), which has been known since 2016 [97, 98]. This popular derivative was the starting point for the formation of additional derivatives (Fig. 3). Substitution of the phenyl ring with 3,4-methylenedioxy has given rise to MDPiHP and fluorine substitution at the *meta* position to 3F-α-PiHP. A derivative very similar to 3F-α-PiHP containing a fluorine substituent in the *meta* position is 3F-α-PHP. Analogs of 3F-α-PHiP and 3F-α-PHP containing fluorine in the *para* position had already been known before 2019 [95, 99–101].

**Fig. 4:**
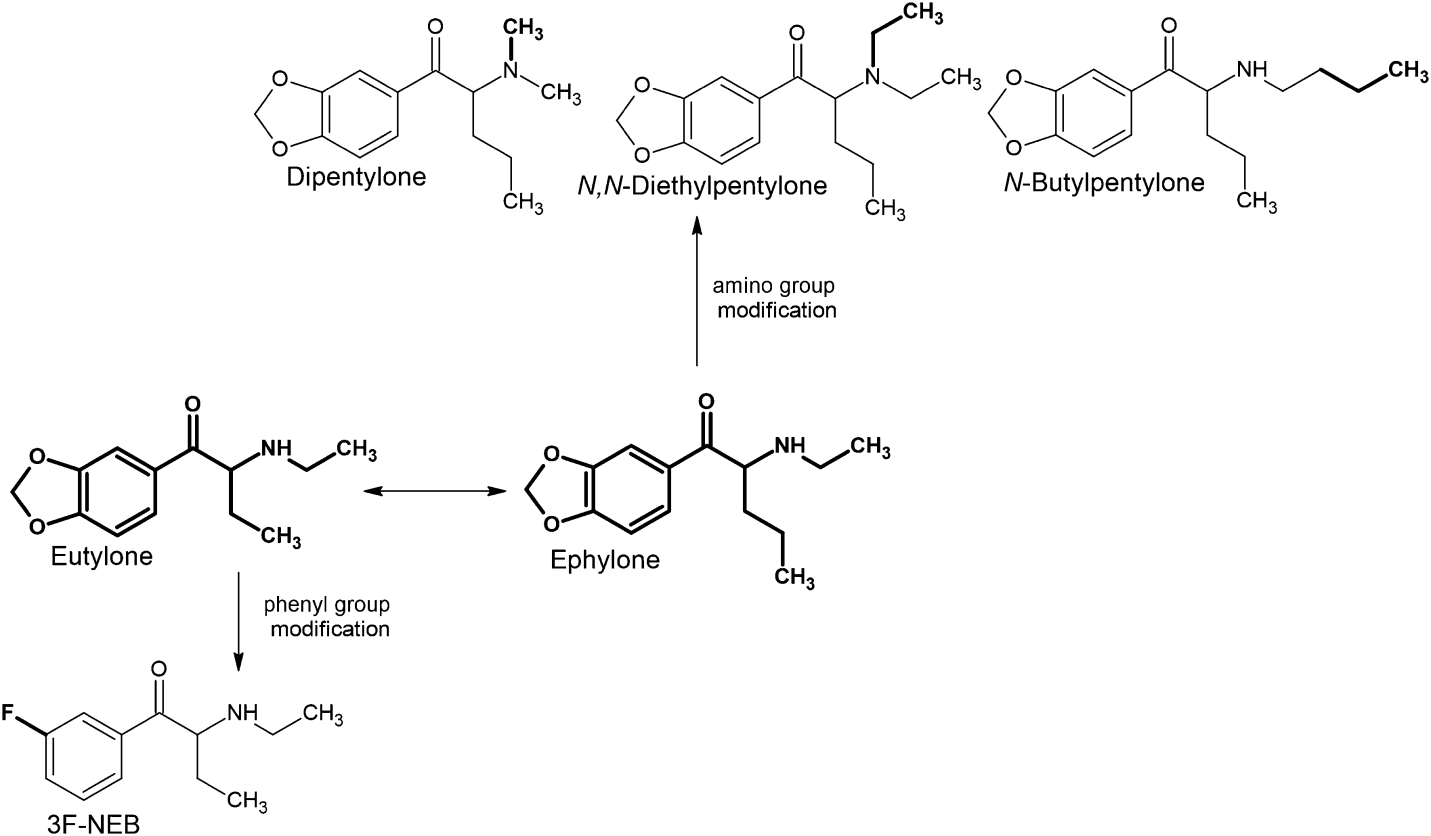
Structures of ephylone and eutylone analogs

Some of the derivatives can be seen as substitutes of another highly popular synthetic cathinone, ephylone (Fig. 4). Modifications within the amine group have resulted in three new derivatives: *N,N*-diethylpentylone (additional *N*-ethyl group), *N*-butylpentylone (elongated *N*-alkyl chain), and dipentylone (*N,N*-dimethyl group). Whereas 3F-NEB, unlike ephylone, has a shorter by one carbon atom alkyl side chain (which makes it more similar to eutylone) with the simultaneous presence of fluorine at the *meta* position of the phenyl ring instead of the 3,4-methylenedioxy moiety.

**Fig. 5:**
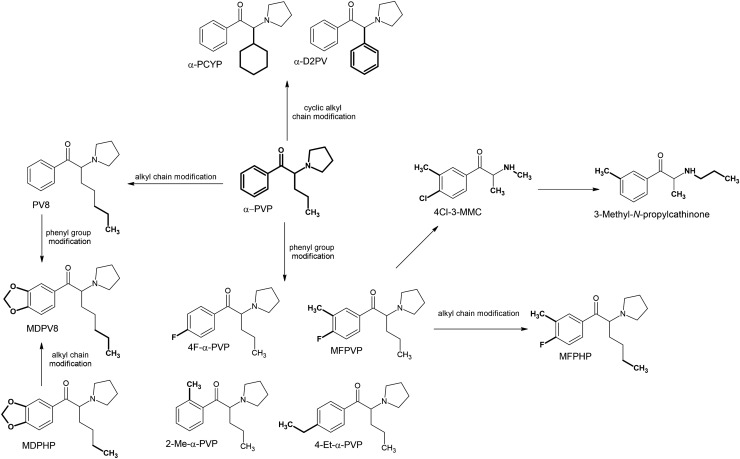
Structures of α-PVP analogs

MDPV8, first identified in 2019, is an analog of other popular synthetic cathinones; PV8 (1-phenyl-2-(pyrrolidin-1-yl)-heptan-1-one) [102, 103], and MDPHP (1-(1,3-benzodioxol-5-yl)-2-(pyrrolidin-1-yl)hexan-1-one) distinguished from them by the presence of a 3,4-methylenedioxy moiety, and by elongated alkyl side chain respectively (Fig. 5). Another extremely popular cathinone is α-PVP, also known as “Flakka” [104, 105], which served as the starting point of other newly developed derivatives (Fig. 5). 4-Et-PVP is a “Flakka” derivative containing an ethyl chain in the *para* position of the phenyl ring, while the 2-Me-PVP contains a methyl group in the ortho position. 4F-3-methyl-α-PVP derivative (MFPVP) is particularly interesting chemically. It is the first synthetic cathinone to contain an alkyl substituent as well as a halogen substituent at the ring. Two more derivatives possessing this characteristic substitution at the phenyl ring were created shortly thereafter. Having a similar structure to MFPVP, MFPHP is the higher homolog having an elongated alkyl chain; and being quite distinct from MFPVP, 4Cl-3MMC has a different halogen (chlorine instead of fluorine), a short alkyl side chain and only a methyl group on the nitrogen atom (Fig. 5). The combination of halogen and alkyl at the phenyl ring may be a new trend, and it is likely that this will be more common in newly developed synthetic cathinones in the future. A derivative quite related to 4Cl-3MMC is 3-methyl-*N*-propylcathinone with the difference being the absence of a chlorine atom and the presence of an extended chain on the nitrogen atom (from methyl to propyl). Other structurally unusual analogs of α-PVP are α-PCYP and α-D2PV, possessing a cyclohexyl and a phenyl ring in place of the alkyl side chain, respectively (Fig. 5). These compounds are rare examples of cathinone derivatives with cyclic groups at the alkyl side chain site.

**Fig. 6:**
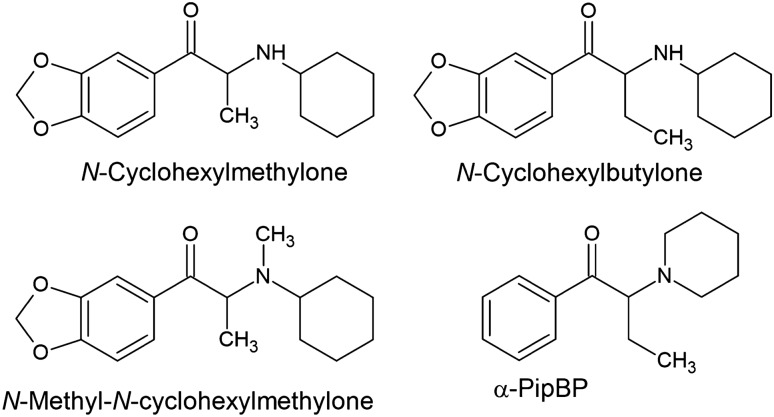
Structures of synthetic cathinones with an unusual substituent attached to the amino group

Other compounds with a cyclohexyl substituent are *N*-cyclohexylmethylone and its *N*-methyl derivative—*N*-methyl-*N*-cyclohexylmethylone, as well as *N*-cyclohexylbutylone, but in their cases each ring is not at the side chain site, but is attached to the amino group (Fig. 6). Another derivative containing an unusual substituent within the amino group is α-PipBP, which contains a six-membered piperidine ring as opposed to a five-membered pyrrolidine ring (Fig. 6).

**Fig. 7:**
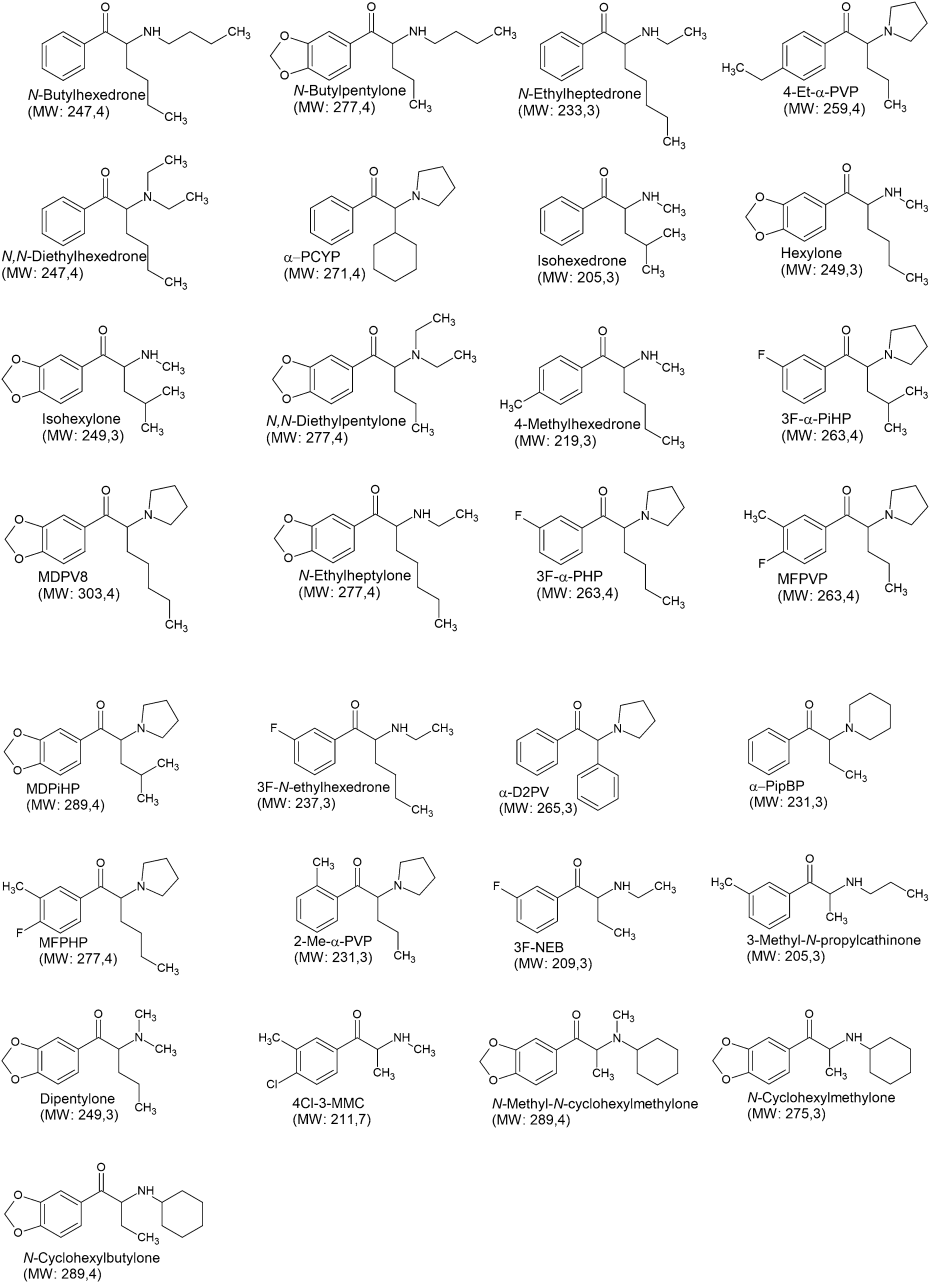
Structures of 29 new synthetic cathinones emerging during 2019–2022. *MW* molecular weight

Of the 29 newly emerged derivatives (Fig. 7), we identified as many as 16 of them with a long (> ethyl) alkyl side chain at the α position, 4 of them with an isobutyl chain, 1 with a phenyl ring, 1 with a cyclohexyl ring and only the remaining 7 derivatives with short alkyl side chains: 4 with a methyl chain and 3 with an ethyl chain. These finding indicate that the trend of synthesizing derivatives with long, extended side chains is continuing and for several years now most of the newly formed derivatives have had such long side chains [106–108].

In terms of the amine group, 15 derivatives are tertiary amines, including 10 with a pyrrolidine ring, 2 with two ethyl chains on the nitrogen atom, 1 with double methyl group on the nitrogen atom, 1 with a double substitution on the nitrogen atom in the form of a methyl and cyclohexyl group, and 1 with a piperidine ring. Among the remaining 14 derivatives, 5 have a short *N*-methyl chain, 4 derivatives have an *N*-ethyl chain, 2 derivatives have a long butyl chain, 1 has a propyl chain and 2 derivatives have an extended *N*-cyclohexyl group. Among the identified compounds, none is a primary amine.

Eleven of the 29 derivatives have a 3,4-methylenedioxy moiety. Four derivatives have an alkyl substituent of the phenyl, two at the *para* position, one at the *meta* position, and one at the *ortho* position. Four derivatives have a fluorine substituent at the *meta* position of the ring and three derivatives contain a rare combination of an alkyl group substitution at the *meta* position with a simultaneous halogen substituent at the *para* position. The remaining seven derivatives have no phenyl ring substituents.

## Pharmacology and toxicity

In general, synthetic cathinones are classified as stimulants or amphetamine-type stimulants [109, 110]. However, the pharmacological effects of individual derivatives are strictly dependent on the type of substituents and their location, and the differences in pharmacological profiles between individual cathinone derivatives are considerable. The range of pharmacological profiles of cathinone derivatives varies from those resembling MDMA and cocaine in action, through cathinone derivatives possessing methamphetamine-like psychostimulant effects, to highly dopaminergic pyrovalerone cathinone derivatives [110, 111]. The structure differs depending on the lipophilicity and steric expansion, which affects the pharmacokinetic aspects, but also the key interaction with monoamine transporters (MATs) in the central nervous system [112, 113]. The main systems involved are dopaminergic, serotonergic and noradrenergic, and the main mechanism of their modulation is the interaction with particular transporters, including dopamine transporter (DAT), serotonin transporter (SERT), and norepinephrine transporter (NET). Synthetic cathinones alter neurotransmitter concentrations by acting as MAT inhibitors or substrates, inducing efflux of endogenous neurotransmitters [110, 111, 114]. Dopaminergic activity is mainly associated with stronger reinforcing effects and abuse liability. In contrast, serotonergic activity is identified with entactogenic effects similar to those of MDMA [111–113, 115, 116]. Pyrrolidine cathinone derivatives are particularly potent dopaminergic, while methylone, similar in structure to MDMA, exhibits serotonergic effects; cathinone derivatives substituted at the phenyl ring in the *para* position also exhibit comparable activity [117]. The strength of action on the noradrenergic system is more or less equal among particular synthetic cathinones; therefore, the main factor distinguishing the action of particular derivatives is selectivity toward DAT and SERT [111]. Depending on the structure of the derivative, the interaction with individual monoaminergic systems is characterized by different strength and selectivity, which translates into desired effects and intoxication symptoms. The main effects of cathinone use include symptoms from the cardiovascular system such as tachycardia and increased blood pressure, and from the nervous system such as euphoria and motor excitation, as well as hyperthermia [7, 118–121]. In addition to psychostimulant effects, some synthetic cathinones may have hallucinogenic properties similar to MDMA. Synthetic cathinones possessing high selectivity toward the serotonin system may lead to a dangerous serotonergic syndrome resulting from excessive activation of 5-HT_2A_ receptors. Most often, however, the appearance of this type of intoxication results from combined drug intoxication, mainly in combination with selective serotonin reuptake inhibitors (SSRIs), monoamine oxidase inhibitors (MAOIs) or serotonin–norepinephrine reuptake inhibitors (SNRIs) [7, 122, 123].

### *N*-Ethylhexedrone, ephylone, and eutylone

Of the synthetic cathinones that emerged in 2019–2021, only a few of them have intoxication case reports. Most information regarding intoxication cases exists for the most popular compounds in 2019–2021—*N*-ethylhexedrone, ephylone and eutylone—while a large proportion of the newly emerged derivatives are their structurally similar analogs. In the absence of direct data, knowledge from SAR studies can be used to predict and estimate the properties of the novel derivatives. In addition, data on pharmacodynamics, pharmacokinetics and toxity are very limited.

*N*-Ethylhexedrone is a pure and potent inhibitor of DAT and NET reuptake, and has low inhibition potency at the SERT, thus its pharmacological profile is similar to pyrrolidine cathinones [124, 125]. *N*-Ethylhexedrone exhibits potential for abuse in rats, and its locomotor stimulant properties are comparable to cocaine and slightly less potent than methamphetamine, while the peak effect lasted longer as compared to cocaine and methamphetamine [126]. According to user reports, however, its desired effect was short-lived, which could lead to dangerous binge dosing [127]. Additionally, *N*-ethylhexedrone has neurotoxic properties and is toxic to microglia [128]. There are numerous descriptions in the literature of fatal [97, 127, 129–133] and non-fatal [100, 127, 134–136] poisoning cases where *N*-ethylhexedrone has been detected in biological material (Table 1). The blood concentrations detected in fatal and non-fatal cases were in the ranges of 4–285 ng/mL and 1–84 ng/mL, respectively, and the poisoning effects described included symptoms typical of sympathomimetic and serotonergic toxidromes. The most common were euphoria, stimulation, agitation, tachycardia, hyperthermia, and aggression, and less commonly were insomnia, paranoia, convulsions, anxiety hallucinations and acute kidney failure. Typical doses are 50–60 mg at one time, but the range can be much wider and dependent on the route of administration [127]. The reported routes of administration include nasal, oral, and inhalation (e-cigarettes), but also rectal administration and intravenous injection. Among the newly emerged drugs that are structurally similar to *N*-ethylhexedrone, one can mention *N*-ethylheptedrone, *N*-butylhexedrone, 3F-*N*-ethylhexedrone, hexylone, 4-methylhexedrone, and *N–N*-diethylhexedrone.

**Table 1:** IUPAC names, reported typical doses, concentrations in biological material in fatal and non-fatal intoxication cases, and reported intoxication symptoms for synthetic cathinones

Compound name	IUPAC name	Typical reported doses [mg]	Conc. in fatal intoxication cases [ng/mL or g]	Conc. in non-fatal intoxication cases [ng/mL]	Reported intoxication symptoms
*N-*Ethylhexedrone	2-(Ethylamino)-1-phenylhexan-1-one	50–60	Blood: 4–285 Urine: 147–1477 Bile: 34 Lung Brain: 5 [97, 127, 129–133]	Blood: 1–84 Urine: 165 [100, 127, 134–136]	Euphoria, stimulation, hyperthermia, tachycardia, vasoconstriction, aggression, dilatated pupils, anxiety, hallucinations, paranoia, acute kidney failure, insomnia, convulsions
Ephylone	1-(1,3-Benzodioxol-5-yl)-2-(ethylamino)-pentan-1-one	10–100	Blood: 1–50,000 Urine: 18,000 [9, 58–62, 133, 143–146]	Blood: 7–149 Urine: 2000–10,700 Oral fluid: 13–1380 [58, 143, 147, 148]	Euphoria, stimulation, tachycardia, hyperthermia, palpitations, delusional state, aggression, psychosis, paranoia, hallucinations, confusion, sleeplessness, cardiac arrest, dissociative effects, acidosis, mydriasis rhabdomyolysis, renal failure
Eutylone	1-(1,3-Benzodioxol-5-yl)-2-(ethylamino)-butan-1-one	50–200	Blood: 1–11,000 Urine: 192,000 Gastric content: 2120 Fat tissue: 1310 [65, 67, 150]	Blood: 1–3600 Urine: 54–18,400 [65, 67, 151–154]	Euphoria, stimulation, tachycardia, hyperthermia, hypertension, agitation, hallucinations, delirium, insomnia, anxiety, paranoia, seizures, cardiac arrest
2-Me-α-PVP	1-(2-Methylphenyl)-2-(pyrrolidin-1-yl)pentan-1-one	20–60	Na	na	Euphoria, stimulation, paranoia, hallucinations, impaired vision and speech, anxiety
MFPVP	1-(4-Fluoro-3-methylphenyl)-2-(pyrrolidin-1-yl)pentan-1-one	10–90	Blood: 26–30 Vitreous humor: 20 [44]	na	Euphoria, stimulation, tachycardia, cardiac arrest
4F-α-PVP	1-(4-Fluorophenyl)-2-(pyrrolidin-1-yl)pentan-1-one	10–30	Blood: 127–145 [160]	Blood: 23–43 Urine: 1–24,600 [159]	Euphoria, stimulation, tachycardia, hyperthermia, hallucinations, paranoia, aggression, pulmonary hemorrhage
MDPV8	1-(1,3-Benzodioxol-5-yl)-2-(pyrrolidin-1-yl)-heptan-1-one	10–80	Na	na	Euphoria, stimulation, tachycardia, hyperthermia, insomnia, paranoia,
MDPHP	1-(1,3-Benzodioxol-5-yl)-2-(pyrrolidin-1-yl)hexan-1-one	5–50	Blood: 7–399 [130, 133, 172, 173]	Blood: 3–140 Urine: 2–5950 [159, 172, 174]	Euphoria, stimulation, tachycardia, hypertension, hyperthermia, anxiety, hallucinations, dizziness, impaired vision, paranoia, aggression
PV8	1-Phenyl-2-(pyrrolidin-1-yl)-heptan-1-one	20–50	Blood: 70–260 Urine: 110–130 [103, 130, 160]	Urine: 2–1270 [159]	Euphoria, stimulation, tachycardia, hypertension, hyperthermia,
α-PCYP	2-Cyclohexyl-1-phenyl-2-pyrrolidin-1-yl-ethan-1-one	20–40	Na	na	Euphoria, stimulation, tachycardia, hyperthermia, restlessness, anxiety, psychosis
α-D2PV	1,2-Diphenyl-2-(pyrrolidin-1-yl)ethan-1-one	20–50	Na	na	Euphoria, stimulation, vasoconstriction, increased libido, anxiety
Dipentylone	1-(1,3-Benzodioxol-5-yl)-2-(dimethylamino)pentan-1-one	na	Blood: 33–970 [62, 77]	na	na

References are shown in brackets

The term "blood" we use here is synonymous with the terms whole blood, plasma and serum. These terms are often used inappropriately in the literature (for example, blood concentration determination was described when in fact serum was tested). Therefore, to simplify and unify, we have used the collective term “blood”. However, it is worth noting that most often in postmortem cases, whole blood was tested, and from living persons, serum was the most commonly tested material

*na* data not available

Ephylone is a pure and potent reuptake inhibitor of DAT. It also shows reuptake inhibition at NET and SERT, albeit to a lesser extent [58]. In rats, ephylone showed locomotor stimulant effects similar to methamphetamine, but was less potent than it and showed a shorter duration of action [137, 138]. In addition, repeated administration of ephylone caused anxiolytic-like and depressive-like effects and aggressiveness in rats [138, 139]. Studies in rodents suggest that ephylone has a high addictive potential [137, 139–141]. Additionally, studies in zebrafish showed its neurotoxicity, cardiotoxicity and developmental toxicity [142]. There are reports of deaths with ephylone detected in biological material [9, 58–62, 133, 143–146], as well as cases of non-fatal poisoning [58, 143, 147, 148]. The blood concentrations of ephylone detected in these cases were 1–50,000 ng/mL and 7–149 ng/mL, respectively (Table 1). Typical doses are 1–100 mg depending on the route of administration, and these are primarily via nasal insufflation, oral consumption, and intravenous injection, and less commonly rectal and sublingual administration. Ephylone intoxication in the reported cases resulted in cardiac arrest, rhabdomyolysis, hyperthermia, tachycardia, psychomotor agitation, palpitations, a delusional state, psychosis, paranoia, and aggressiveness, among others. Many newly emerged drugs are analogs and substitutes of ephylone, including *N*-butylpentylone, *N,N*-diethylpentylone, *N*-ethylheptylone, and dipentylone.

Eutylone, a popular alternative to ephylone, showed hybrid activity; it was a partial substrate for SERT and at the same time a pure uptake inhibitor toward DAT and NET. Based on its pharmacology, eutylone has a greater potential for abuse and dose-dependent locomotor stimulation in rodents as compared with its structural isomers pentylone and dibutylone [67, 149]. As a result of the popularity of this compound, there are many reports of fatal [65, 67, 150] and non-fatal [65, 67, 151–154] poisonings, which were confirmed by detecting eutylone in blood. The concentration ranges were 1–11,000 ng/mL in fatal cases and 1–3600 ng/mL in non-fatal cases (Table 1). Typical doses used start at around 50 mg when administered via the nasal route; when taken orally, the typical dose is higher up to 200 mg [67]. Other routes of administration were also reported, mainly smoking (e-cigarettes), intravenous injection, and rectal administration. User-reported desired effects include euphoria, and stimulation, while the adverse effects include mainly tachycardia, hyperthermia, agitation, and hypertension, but also hallucinations, delirium, paranoia, anxiety, seizures, cardiac arrest, and rhabdomyolysis.

### 2-Me-α-PVP

2-Me-α-PVP also known as *ortho*-pyrovalerone is a new synthetic cathinone that has been extensively commented on drug user forums in 2021. This derivative was already synthesized in 2006 and tested along with other pyrovalerone analogs[155]. It differs from pyrovalerone in the position of the methyl group at the phenyl ring (4-methyl vs. 2-methyl). 2-Me-α-PVP is similarly to pyrovalerone a potent DAT and dopamine reuptake inhibitor. These compounds also exhibit norepinephrine reuptake inhibition, and are inactive toward SERT. However, 2-Me-α-PVP shows about twofold weaker effects on DAT and NET as compared to the 4-methyl analog [155]. According to drug user forum, typical low doses were about 20–30 mg, and high doses start at about 50 mg. Dosage can differ depending on the route of administration and these include smoking (e-cigarettes), oral consumption, intravenous injection, and snorting. The most commonly described symptoms of use included stimulation, euphoria, but also adverse effects such as paranoia, hallucinations, impaired vision and speech, and panic attacks (Table 1). Users indicated that the drug had a very strong addictive effect, comparing it to that of the α-PVP [156, 157].

### MFPVP

In January 2022, Hobbs et al. [44] described the first case of fatal poisoning of a 30-year-old man solely as a result of MFPVP toxicity. The postmortem concentrations found were 26 ng/mL in femoral blood, 30 ng/mL in heart blood and 20 ng/mL in vitreous humor. MFPVP was also detected qualitatively in urine. Manifestations of intoxication were ventricular tachycardia and cardiac arrest, while the autopsy revealed pulmonary edema, cardiomegaly, and cerebral edema. The desired effects reported by users in online forums [27, 158] included increased energy and euphoria (Table 1). The doses that they use are typically in the range of 10–90 mg, depending on the route of administration, which include nasal insufflation, smoking (e-cigarettes), and intravenous injection. The duration of action described by users was 3–5 h. MFPVP is the first known cathinone derivative to be simultaneously substituted with a halogen and an alkyl substituent, and therefore no studies on the pharmacology and toxicity of this type of derivative exist yet. Structurally, MFPVP is most similar to 4F-α-PVP (1-(4-fluorophenyl)-2-(pyrrolidin-1-yl)pentan-1-one), which differs only in the absence of a methyl group at the *meta* position of the phenyl ring. In non-fatal intoxication cases 4F-α-PVP concentrations were 1–24,600 ng/mL in urine and 23–43 ng/mL in serum [159]. 4F-α-PVP was also found in a multiple cathinone intoxication case at concentrations of 145 ng/mL in heart blood and 127 ng/mL in femoral vein blood. A 20-year-old male showed unusual behavior before his death and was found naked in his own room with his head through a wall [160]. Interestingly a case of diffuse alveolar hemorrhage following 4F-α-PVP and *N*-ethylpentedrone inhalation was also reported [161]. Pyrrolidine derivatives show high potency even at low doses, and a typical dose for 4F-α-PVP was 10–20 mg [162]. 4F-α-PVP showed less cytotoxicity than its more lipophilic analogs with extended aliphatic side chains, while at the same time showing greater stimulatory effects at the same doses [163, 164]. However, the presence of fluorine at the *para* position in the case of pyrrolidine cathinone derivatives favored increased cytotoxicity [165]. In addition, *para*-halogenation was associated with a slightly weaker potency with DAT and dopamine reuptake inhibition and, at the same time, stronger serotonergic effects, as well as probable anxiogenic effects [124, 166, 167].

### MDPV8

MDPV8 is a novel compound and a higher homolog of MDPHP and at the same time a methylenedioxy derivative of PV8, a substance that is Schedule I in the USA. Unlike MDPV8, these derivatives were more widely studied, and cases of poisoning with these substances were described. To the best of our knowledge, there is no information regarding detected blood concentrations in non-fatal and fatal MDPV8 poisonings, although there are reports of detection of this compound in peripheral blood in postmortem toxicology cases [38]. Additionally, α-PHP analogs including MDPV8 were investigated in 2020 for their activity against human muscarinic receptors [168]. The concentrations found in cases of fatal PV8 intoxications were 70–260 ng/mL in blood and 110–130 ng/mL in urine (Table 1). PV8 was also determined in the liver and kidney, and autopsies showed pulmonary and cerebral edemata, and pathological changes in the heart [103, 130, 160]. Urinary concentrations of 2–1270 ng/mL were demonstrated in cases of non-fatal PV8 intoxications [159]. Common single doses of PV8 are between 20 and 40 mg, but doses as high as approximately 400 mg were also reported [8, 162]. Pyrrolidine cathinone derivatives with elongated aliphatic side chains such as PV8 were shown to have high cytotoxicity, which was further increased by the presence of the methylenedioxy grouping (also present in the new MDPV8), and also had fewer psychostimulant effects when taken at the same doses, which may induce users to use them at higher doses, further increasing their harmfulness [163, 165, 169–171]. Concentrations found in cases of fatal MDPHP poisoning were 7–399 ng/mL in postmortem blood [130, 133, 172, 173], while concentrations of 2–5950 ng/mL in urine and 3–140 ng/mL in blood were detected in non-fatal intoxications (Table 1) [159, 172, 174]. Typically reported single dosages of MDPHP ranged from 5 to 50 mg [172], and reported symptoms of intoxication include hypertension, tachycardia, euphoria, anxiety, dizziness, blurred vision, insomnia, focus enhancement, breathing difficulties, paranoia, elevated temperature, psychosis, aggressiveness, thorax pain, and hallucinations [159, 172, 174]. Due to the similarity in structure of MDPV8 to MDPHP and PV8, it can be hypothesized that when taken, it exhibits similar concentrations and causes similar symptoms.

### α-PCYP

Of the newly emerged synthetic cathinones we identified, one of the compounds most frequently commented on in drug user forums has been α-PCYP [27]. Before hitting the market, α-PCYP was one in a series of α-PVP analogs tested in 2015 for pharmacological effects in rat brain synaptosomes [28]. The presence of a lipophilic and extremely bulky cyclohexyl ring in place of the alkyl side chain means that α-PCYP, as compared with the popular α-PVP having a propyl side chain, exhibited up to twofold stronger interaction with DAT. This ability may translate into stronger dopaminergic stimulation and higher addictive potential [28]. User reports suggested that the most common single doses were 20–40 mg and resulted in a 2–5 h duration of action. The dose and duration of the effect depended on the route of administration. The most commonly reported desired effects include stimulation and euphoria, while the described adverse effects include restlessness, anxiety, psychosis, tachycardia, and hyperthermia (Table 1) [175–179]

### *N*-Butylpentylone and *N*-butylhexedrone

*N*-Butylpentylone is an analog of ephylone with an extended *N*-alkyl chain (from *N*-ethyl to *N*-butyl), whereas *N*-butylhexedrone is a higher analog of the popular *N*-ethylhexedrone. We have described these popular analogs in the previous section. Although these novel compounds have been detected repeatedly in many countries, we found no literature reports describing intoxication cases [18, 20, 22]. In addition, the pharmacological effects of these compounds have also not been studied. SAR studies suggest that a more bulky and lipophilic substitution on the amino group promotes increased potency of dopamine reuptake inhibition which translates into strong stimulant effects and large rewarding properties. However, this is not a linear relationship: while the change from an *N*-methyl to an *N*-ethyl chain increases potency, substituting the pyrrolidine ring with a larger piperidine ring or with an *N,N*-diethyl substituent decreases potency [140, 180, 181]. Hence, it is likely that *N*-butyl analogs have fewer stimulatory properties than their *N*-ethyl counterparts, but this needs to be confirmed in further studies.

### α-D2PV

The presence of a phenyl ring in place of the alkyl side chain significantly distinguishes α-D2PV from other synthetic cathinones. α-D2PV can be regarded as an analog of α-PVP, but it also structurally resembles other research drugs from the group of 1,2-diphenylethylamines, which have gained popularity as alternatives to arylcyclohexylamines, and which lack the β-ketone group characteristic of synthetic cathinone [182]. A popular compound belonging to this group of research drugs is diphenidine (1-(1,2-diphenylethyl)piperidine), which, in addition to the absence of the ketone grouping, is distinguished from α-D2PV by the presence of a piperidine ring in place of the pyrrolidine ring [183]. DPPy (1-(1,2-diphenylethyl)pyrrolidine) is an analog of α-D2PV that differs from it only in the absence of a ketone group [182, 184]. The action of compounds in the 1,2-diphenylethylamine group is mainly associated with their antagonistic effects on *N*-methyl-d-aspartate receptors (NMDARs), resulting in dissociative effects. However, they are known to interact with other molecular targets for example, monoamine neurotransmitter transporters [182, 184–187]. There are reports of both fatal and non-fatal diphenidine poisoning [182, 183, 187, 188]. The most commonly described symptoms of poisoning are hypertension, tachycardia, anxiety, altered mental status, and hallucinations. Although the presence of a β-ketone grouping distinguishes α-D2PV from 1,2-diphenylethylamines, it is likely that this compound partially shares the effects of compounds from the 1,2-diphenylethylamine group and, at the same time, exhibit partially different effects from the other cathinone derivatives. However, this hypothesis needs to be confirmed by studies. Some users of drug forums do indeed indicate that α-D2PV has a slightly different effect from the other synthetic cathinones known to them, while at the same time the symptoms they most frequently describe are moderate euphoria, stimulation, increased libido, vasoconstriction, anxiety, and the inability to focus (Table 1). The typical dose is 20–50 mg; the duration of action is estimated by users to be around 2 h and the most common routes of administration include inhalation (e-cigarettes), intravenous injection, and nasal insufflation [189–191].

### Dipentylone

In April 2022, the CFSRE issued a public health alert regarding the increased frequency of detection of dipentylone intoxication [77]. Dipentylone was detected in 32 toxicology cases across the USA, with detected concentrations in postmortem blood ranging from 33 to 970 ng/mL (Table 1). On drug user forums there were relatively few threads dedicated to this substance. Dipentylone is described by users as a low-potency substance with the suspicion that it acts as a prodrug of the mono-*N*-methyl analog—pentylone. Indeed, pentylone may be a major metabolite of dipentylone, as one of the main mechanisms of synthetic cathinone metabolism is *N*-dealkylation [4, 192, 193]. Apart from the data made available by the CFSRE, there is so far only a single report of a polydrug intoxication case where dipentylone was detected among others [62]. Nonetheless, this compound has been being the subject of several studies on its pharmacology. Dipentylone, as compared with MDPV, had a fivefold lower affinity toward DAT [180] and about tenfold higher selectivity toward DAT as compared with SERT [124]. Additionally, a study in rats showed that the locomotor stimulant properties of dipentylone were similar in potency to cocaine and less potent than methamphetamine; at the same time, the effect lasted as long as methamphetamine and longer than cocaine. However, the doses required to elicit stimulant effects were relatively high when it was compared with other synthetic cathinones, meaning that there is an increased risk of side effects when used recreationally [126]. The high doses required may be partly explained by the low permeability of dipentylone across the blood–brain barrier (BBB). The slightly different structural isomer ephylone (*N,N*-dimethyl versus *N*-ethyl) had an almost 14-fold higher BBB permeability, demonstrating the profound effect of even small structural changes on pharmacokinetic properties [194]. However, it is important to note that BBB permeability is generally not a factor in the potency of a compound.

## Conclusions

In recent years, many novel compounds from the synthetic cathinone group have appeared on the illicit drug market. Most of these substances have been poorly or not at all studied for their pharmacological properties and toxicity. Fatal poisoning incidents with these substances demonstrate the high risk posed by these agents. Hence, there is the need for (1) further research on the properties of these compounds and (2) continued improvement of early warning systems. Some of the newly identified emerging synthetic cathinones are already posing a threat to public health and safety, and some, although they do not appear to be widespread now, may gain significance in future years.
